# Influence of the dual ABCB1 and ABCG2 inhibitor tariquidar on the disposition of oral imatinib in mice

**DOI:** 10.1186/1756-9966-28-99

**Published:** 2009-07-10

**Authors:** Erin R Gardner, Nicola F Smith, William D Figg, Alex Sparreboom

**Affiliations:** 1 Clinical Pharmacology Program, SAIC-Frederick, Inc, NCI-Frederick, Frederick, Maryland, 21702, USA; 2 Clinical Pharmacology Program, Medical Oncology Branch, Center for Cancer Research, National Cancer Institute, Bethesda, Maryland, 20892, USA; 3 Department of Pharmaceutical Sciences, St Jude Children's Research Hospital, Memphis, Tennessee, 38105, USA

## Abstract

**Background:**

Imatinib, a tyrosine kinase inhibitor currently approved for treatment of several malignancies, has been shown to be a substrate for multiple efflux-transporter proteins, including ABCB1 (P-glycoprotein) and ABCG2 (BCRP). The effect of inhibiting these transporters on tissue exposure to imatinib remains unclear.

**Objective:**

To assess the role of these transporters on drug disposition, 50 mg/kg imatinib was administered to Balb/C mice, 30 minutes after receiving tariquidar (10 mg/kg), an inhibitor of both ABCB1 and ABCG2, or vehicle, via oral gavage.

**Methods:**

Quantitative determination of imatinib in mouse plasma, liver and brain was performed using a newly-developed and validated liquid-chromatography-mass spectrometric method. Results: Exposure to imatinib was 2.2-fold higher in plasma, liver and brain in mice that received tariquidar, as compared to those that received the vehicle (P = 0.001). The peak plasma concentration did not increase substantially, suggesting that tariquidar is affecting the distribution, metabolism and/or excretion of imatinib, rather than absorption. Though tariquidar increased the absolute exposure of imatinib, the brain-to-plasma ratio of imatinib was unaffected.

**Conclusion:**

This study suggests that intentional inhibition of ABCB1 and ABCG2 function at the blood-brain barrier is unlikely to significantly improve clinical outcome of imatinib with currently used dosing regimens.

## Background

Imatinib mesylate is an orally administered tyrosine kinase inhibitor, currently FDA approved for the treatment of Philadelphia chromosome-positive chronic myeloid leukemia (targeting Brc-Abl) and unresectable and/or metastatic malignant gastrointestinal stromal tumors (targeting c-KIT) [1]. This agent is also currently under intensive investigation in other tumor types, most notably as a single agent or in combination with hydroxyurea for the treatment of gliomas. However, there has been limited clinical success reported to date [2,3].

Imatinib was initially determined to be a substrate for ABCB1 (P-glycoprotein) *in vitro *[4]. Subsequently, it was demonstrated that the *in vivo *distribution of imatinib is limited by ABCB1-mediated efflux, resulting in limited brain penetration [5]. More recently, positron emission topography studies with [*N*-^11^C-methyl]-imatinib have confirmed limited brain penetration in primates [6]. However, ABCB1 is not the sole transporter expressed in the blood-brain barrier that may limit the brain distribution of imatinib. In particular, imatinib is both an inhibitor [7] and substrate [8] of ABCG2 (BCRP). Experiments comparing the plasma and brain pharmacokinetics of imatinib following i.v. administration of radiolabeled drug to wild-type, *Abcb1 *knockout and *Abcg2 *knockout mice have confirmed a role of these transporter proteins in limiting brain exposure [9].

The potential influence of these efflux transporters is not limited to brain exposure. For example, ABCB1 and ABCG2 are also highly expressed in the small intestine, bile canaliculi of the liver and numerous other normal tissues [10,11]. In addition, expression of these proteins in human tumors has been associated with development of multidrug resistance [12]. Furthermore, *in vitro *studies have suggested that long-term treatment with imatinib leads to increased expression of both ABCB1 and ABCG2, resulting in decreased intracellular drug accumulation [13]. As such, it is of great interest to identify and characterize inhibitors of ABCB1 and ABCG2 *in vivo *that could potentially be used to intentionally alter the pharmacokinetics of and/or improve response to therapy with anticancer ABCB1 and ABCG2 substrates [11].

Several transporter inhibitors have previously been evaluated in preclinical models, including the ABCB1 inhibitors valspodar and zosuquidar, the ABCG2 inhibitor pantoprazol and the dual ABCB1/ABCG2 inhibitor elacridar [9,14]. Tariquidar, an orally available anthranilic acid derivative, has been shown to be an inhibitor of both ABCB1 and ABCG2 [15]. It is currently in clinical trials evaluating its utility as an inhibitor of ABCB1, in an effort to overcome resistance associated with anticancer chemotherapy [16]. Here, we evaluated the effect of tariquidar on the disposition of imatinib in mice, in order to provide a pharmacokinetic rationale for attempts to improve the agent's low brain penetration.

## Methods

### Chemicals and reagents

Imatinib mesylate was supplied by Novartis (East Hanover, NJ). Tariquidar was supplied by Dr. Susan Bates (NCI, Bethesda, MD). Glucose, harmine, absolute ethanol and ammonium acetate were purchased from Sigma-Aldrich (St. Louis, MO). Formic acid (98%) was obtained from Fluka (through Sigma-Aldrich). Methanol (J.T. Baker, Phillipsburg, NJ) was of HPLC grade. Deionized water was generated with a Hydro-Reverse Osmosis system (Durham, NC) connected to a Milli-Q UV Plus purifying system (Billerica, MA). Blank mouse plasma was purchased from Innovative Research (Southfield, MI).

### Sample Preparation

Unknown and quality control (QC) plasma samples were thawed at room temperature, vortex mixed for 20 seconds, and 100 μL were transferred to a polypropylene centrifuge tube. For analysis of unknown tissue samples, approximately 100 mg of tissue were accurately weighed and water added (5 μL per mg). After vortex-mixing, samples were homogenized using a PowerGen 125, while kept on ice. One hundred μL of homogenate was transferred to a clean polypropylene centrifuge tube for further processing. To each tube, including calibrators (10, 25, 50, 100, 500 and 1000 ng/mL) and QC samples (30, 450, 800 and 18,000 ng/mL), 250 μL of methanol (containing 25 ng/mL of internal standard, harmine) was added. All tubes were capped, vortex-mixed for 5 min and then centrifuged for 5 min at 18,000 × *g*. Following centrifugation, the supernatant was transferred to a vial for injection. Either 5 or 10 μL of the supernatant was injected for tissue or plasma samples, respectively. Calibration curves and QC samples were prepared in both brain and liver, for tissue sample analysis. The working ranges for liver and brain were 0.125–100 and 0.125–25 ng/mL, respectively.

### Equipment

High performance liquid chromatography was carried out on an Agilent 1100 system (Agilent Technology, Palo Alto, CA), coupled with a single-quadrupole mass spectrometer, utilizing electrospray ionization in positive mode. Samples were cooled to 4°C in a thermostated autosampler and the column compartment, containing a Waters SymmetryShield RP8 column (2.1 × 50 mm, 3.5 μm), was maintained at 35°C. Samples were eluted using a gradient mobile phase, comprised of 10 mM ammonium acetate with 0.1% formic acid and methanol, running at a flow rate of 0.35 mL/min for 10 min, including re-equilibration. Mass spectrometric conditions were as follows: fragmentor, 150 V; gain, 2; drying gas flow, 10 L/min; drying gas temperature, 300°C; nebulizer pressure, 40 psi; and capillary voltage, 1500 V. Selected-ion monitoring was accomplished at *m/z *494.2 for imatinib and *m/z *213.1 for the internal standard. The chromatographic data were acquired and analyzed using the Chemstation software package (Agilent).

### Validation procedures

Calculation of accuracy and precision was carried out according to procedures reported in detail previously [17]. Calibration samples were prepared fresh each day in the relevant matrix and frozen QC samples were defrosted and analyzed. A 1/x^2 ^weighting scheme was employed in the generation of standard curves to account for concentration dependent variance. Detector response for plasma was found to be linear in the imatinib concentration range of 10–1000 ng/mL. Plasma accuracy and precision were evaluated with QC samples. Overall, the assay was found to be accurate (deviation of less than 10% for QCs) and precise (within run precision <10%, between run precision <12.6%) for plasma, liver, and brain.

### Animals

All experiments were performed on six-week old, male, Balb/C mice obtained from Charles River Laboratories (Wilmington, MA). The mice weighed approximately 15 to 20 g at the time of study. All mice were allowed unlimited access to water and rodent chow prior to, and during the experiment. Blank mouse liver and brain samples were harvested from surplus mice following euthanasia. NCI-Frederick is accredited by AAALAC International and follows the Public Health Service Policy for the Care and Use of Laboratory Animals. Animal care was provided in accordance with the procedures outlined in the "Guide for Care and Use of Laboratory Animals" (National Research Council; 1996; National Academy Press; Washington, DC). The study design and protocol were approved by the NCI Animal Care and Use Committee (Bethesda, MD).

### Experimental Design

Imatinib was dissolved in sterile water to make a 10 mg/mL dosing solution. Tariquidar was prepared as a 2 mg/mL solution in water with 5% glucose. Mice received either 10 mg/kg tariquidar or the vehicle (5 mL per kg weight) [15] 30 minutes prior to 50 mg/kg of imatinib [18]. All compounds were administered via oral gavage. At each time point, three mice in each treatment group were anesthetized with isoflurane, and bled via cardiac puncture into a tube containing sodium heparin as an anticoagulant. Blood samples were centrifuged at 18,000 × *g *for 5 minutes at 4°C, the plasma layer transferred to a cryovial and frozen. Following euthanasia by cervical dislocation, brain and liver tissues were excised and snap-frozen. All samples were stored at -80°C until the time of analysis.

### Statistical and pharmacokinetic analysis

Concentration-time data were evaluated using a non-compartmental approach, with WinNonlin 5.0 (Pharsight, Mountain View, CA), using the mean concentration (n = 3) at each time point. The peak plasma concentration (C_max_) and the time to peak plasma concentration (T_max_) are reported as observed values. The area under the curve (AUC) was calculated using the linear trapezoidal method from time zero to the time of the last sample with measurable drug concentration. To allow for direct comparison between the two groups and characterization of the terminal phase for the imatinib alone arm, the 24-hour plasma and liver samples, along with the 4-hour brain samples were estimated at LLQ/2, as drug was detectable, but measured concentrations were below the limit of quantitation. Bailer's method was employed to assess the variance, allowing for comparison of exposure between the two dose groups. The significance of the difference in AUC was evaluated by a Z-test. Brain concentrations were corrected for drug in the brain vascular space, by subtracting 1.4% of the plasma concentration from the measured brain concentration for each animal [5]. Brain-to-plasma concentration ratios were calculated for each animal at the 2-hour time point, and the groups compared using a t-test. All statistical tests were performed in Microsoft Excel 2004 (Redmond, WA). P-values < 0.05 were considered significant.

## Results

**Figure 1:**
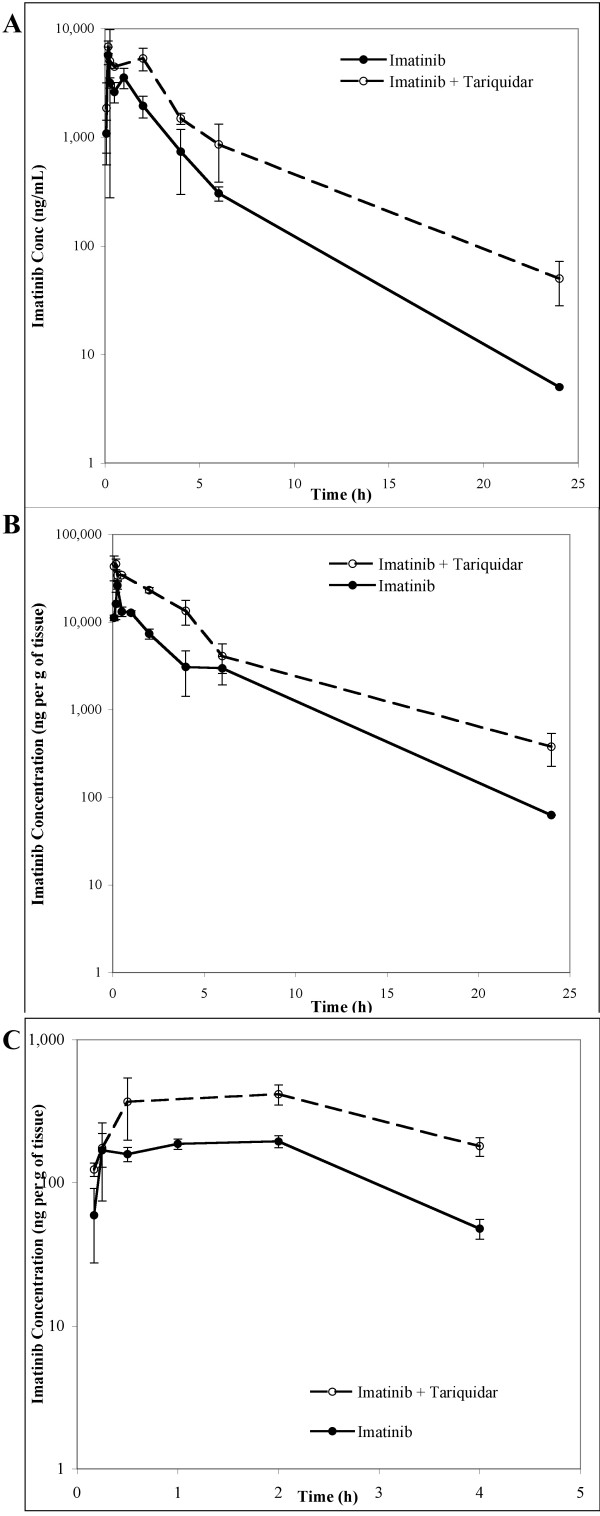
**Concentration-time profiles of imatinib in A. plasma, B. liver and C. brain, for the imatinib plus vehicle group (solid line) and the imatinib plus tariquidar group (dashed line)**. Error bars for each timepoint represent the standard error.

The administration of oral tariquidar 30 minutes prior to an oral dose of imatinib resulted in a significant increase in systemic exposure to imatinib (Table 1; Figure 1). Tariquidar increased the peak plasma concentration of imatinib by 19% (6,813 ± 1,548 vs 5,711 ± 1,472 ng/mL, P = ns), with no apparent change in the rate of absorption, as judged from the similar times to peak concentration (0.17 hours). In contrast, the AUC_0–24 _for imatinib was 2.2-fold higher in mice pretreated with tariquidar compared to the vehicle (26,725 vs 12,168 hr*ng/mL, P = 0.001). In liver tissue, tariquidar increased the peak concentration by 75% (46,139 vs 26,280 ng/g) and the AUC_0–24 _was also 2.2-fold higher (153,209 vs 68,331 hr*ng/mL, P < 0.00001). The maximal corrected concentration of imatinib achieved in brain tissue was 114% higher in the imatinib plus tariquidar group (417 vs 195 ng/g), and the AUC_0–4 _was 2.2-fold higher (417 vs 195 hr*ng/mL, P = 0.00002). No imatinib was detectable in the brain within the first 5 minutes after administration in either group, and the maximal brain concentration was observed after two hours in both groups. The brain-to-plasma ratio of imatinib 2 hours after administration did not differ significantly between the two groups (P = 0.83), and similar brain-to-plasma AUC_0–4 _ratios were observed for each group (0.070 for imatinib plus vehicle *versus *0.078 for imatinib plus tariquidar). In addition, the liver-to-plasma AUC_0–24 _ratios did not differ significantly between the two groups.

**Table 1:** Pharmacokinetics of imatinib in Balb/C mice in the presence and absence of tariquidar

	**Imatinib alone**		**Imatinib + Tariquidar**			
			
Plasma	Mean	SD	Mean	SD	Fold Change	P-value
C_max _(ng/mL)	5,710.5	1,472.3	6,813.2	1,547.9	1.19	-
T_max_ (hr)	0.17	-	0.17	-	-	-
AUC_0–24_ (hr*ng/mL)	12,167.5	-	26,724.6	-	2.20	0.001
Liver	Mean	SD	Mean	SD	Fold Change	P-value

C_max_ (ng/g)	26,279.7	4,560.2	46,139.1	11,000.6	1.76	-
T_max_ (hr)	0.25	-	0.17	-	-	-
AUC_0–24_ (hr*ng/g)	68,330.8	-	153,209.2	-	2.24	< 0.00001
Brain	Mean	SD	Mean	SD	Fold Change	P-value

C_max_ (ng/g)	194.7	27.2	417.0	116.6	2.14	-
T_max_ (hr)	2	-	2	-	-	-
AUC_0–4_ (hr*ng/g)	574.23	-	1,277.7	-	2.23	0.00001

## Discussion

The current study indicates that administration of the dual ABCB1 and ABCG2 inhibitor tariquidar results in a statistically significantly increase in plasma, liver and brain exposure to imatinib. Since imatinib is known to have very high bioavailability (approximately 98%) [1], it is likely that the difference in plasma AUC is due to modified distribution and/or elimination of the drug, rather than a change in the extent of intestinal absorption. This hypothesis is supported by the fact that tariquidar increased the peak plasma concentration of imatinib by less than 20% and this change was not statistically significant. As expected, there was also no apparent change in the rate of absorption. Considering that imatinib is effluxed by both ABCB1 and ABCG2, the almost complete bioavailability may seem somewhat surprising. However, it is possible that the high concentrations of imatinib in the gut are actually leading to localized inhibition of these transporters, as has been suggested by inhibition data [7].

Inhibition of ABCB1 and ABCG2 by tariquidar may also alter the extent of imatinib metabolism. Bihorel *et al*. noted an increase in plasma concentrations of imatinib metabolites in both *Abcb1a/1b *knockout and *Abcg2 *knockout mice; however, co-administration of elacridar, another dual ABCB1 and ABCG2 inhibitor, did not alter the concentrations of imatinib metabolites [19]. Therefore, it is unclear whether this observation may arise due to a compensatory mechanism in the knockout mice.

The brain-to-plasma concentration ratio of imatinib 2 hours after administration was not significantly affected by tariquidar. In addition, the AUC_0–4 _ratio for brain-to-plasma was similar in the presence or absence of tariquidar. This suggests that, rather than modifying the blood-brain barrier directly, tariquidar may simply be increasing plasma concentrations of the drug, leading to saturation of these efflux transporters at this site. The AUCs of imatinib in plasma and both of the tissues studied were 2.2-fold higher following pre-treatment with tariquidar. If modulation at the blood-brain barrier were occurring, independent of increased plasma concentrations of drug, it was hypothesized that the brain accumulation would be greater, not merely the same, as the increase in plasma.

Initial comparison of the inhibitory effects of tariquidar toward ABCB1 and ABCG2, as compared to elacridar, in the context of imatinib disposition, may suggest that tariquidar is less potent, in spite of previously published data that supports the opposite [20]. Specifically, elacridar has been shown to result in a 9.3-fold increase in the brain-to-plasma concentration ratio, as compared to administration of imatinib alone [14]. However, those experiments utilized significantly lower doses of imatinib as compared to the present study (12.5 *versus *50 mg/kg), and the absolute concentrations of drug in brain were not stated. Hence, it is possible that the higher imatinib dose utilized in the current study results in higher plasma concentrations of drug and, therefore, saturation of drug efflux at the blood-brain barrier. In this context, it is particularly noteworthy that single dose plasma pharmacokinetics of imatinib in humans at the recommended oral dose of 400 mg per day results in overall drug exposure that is very similar to that found in the current study for mice (24.8 ± 7.4 *versus *26.3 ± 4.6 h* μg/mL) [1].

Direct comparison between this study and prior experiments investigating the effect of ABC transporter inhibitors on imatinib pharmacokinetics are difficult due to a variety of reasons. The current study employed oral dosing at 50 mg/kg of imatinib, in an effort to closely mimic the clinical situation, whereas Breedveld et al. administered 12.5 mg/kg of imatinib intravenously (in combination with elacridar) [9]. These authors also examined the effect of oral pantoprazole on the pharmacokinetics of 100 mg/kg oral imatinib [9]. Though the increase in brain exposure to imatinib was reported to be higher with oral administration, as compared to i.v., this was only measured at 4 hours post-imatinib, and the analysis was based only on measurement of total radioactivity. As such, it is impossible to determine whether the higher radioactivity in the brain is due to the parent drug only or the parent drug plus metabolites.

Mistry et al. have demonstrated that the inhibitory effect of tariquidar on drug efflux *in vitro *persists for over two hours [15]. In healthy volunteers, a dose of 2 mg/kg i.v. or ≥ 200 mg orally, resulted in 100% inhibition of ABCB1 in CD56+ lymphocytes for over 24 hours. The maximal effect was observed between 2 and 6 hours after administration of tariquidar. In the current study, tariquidar was administered 30 minutes prior to imatinib administration in an effort to ensure sufficient distribution and inhibitory effects.

## Conclusion

In conclusion, oral administration of tariquidar prior to oral imatinib resulted in increased imatinib exposure in plasma and tissues, including brain. The increase in brain exposure appears to be directly related to the increase in plasma concentrations of the drug, at a dose comparable to that used clinically. This further substantiates the possibility that ABC transporters localized in the blood brain barrier are more resistant to inhibition than at other tissue sites such as the intestine and liver [20]. In a clinical setting, the currently observed increase in plasma AUC could result in increased toxicity, as has been observed previously with the use of ABCB1 inhibitors [21]. One strategy that has been employed is dose reduction prior to combining the ABCB1 and ABCG2 substrate with the transporter inhibitor to avoid this toxicity.

Based on our findings, simply doubling the dose of imatinib without addition of an inhibitor would likely result in a similar increase in overall brain exposure, due to increased plasma concentrations of drug. It should be anticipated that inhibition of ABCB1 and ABCG2 function at the blood-brain barrier will not result in a selective increase in brain penetration or improved clinical outcome, beyond that achieved through dose-escalation.

## Competing interests

The authors declare that they have no competing interests.

## Authors' contributions

ERG participated in study design, performed analytical and animal experiments, carried out all statistical analyses and drafted the manuscript. NFS participated in study design and performed animal experiments. WDF participated in study design and helped to draft the manuscript. AS participated in study design, performed animal experiments and helped to draft the manuscript. All authors approved the final manuscript.
